# Measuring Early Childhood Development Among 4–6 Year Olds: The Identification of Psychometrically Robust Items Across Diverse Contexts

**DOI:** 10.3389/fpubh.2021.569448

**Published:** 2021-02-03

**Authors:** Adelle Pushparatnam, Diego Armando Luna Bazaldua, Alaka Holla, João Pedro Azevedo, Marguerite Clarke, Amanda Devercelli

**Affiliations:** World Bank Group, Washington, DC, United States

**Keywords:** early childhood education, caregiver report, direct assessment, cross-cultural, psychometric, early literacy and numeracy skills, social-emotional competencies, executive function (EF)

## Abstract

The last 15 years have seen an explosion of measurement tools for assessing the development of young children in low- and middle- income countries. This paper builds on and contributes to that literature by identifying a core set of caregiver-report items and a core set of direct assessment items that measure key developmental domains for children aged 4–6 (48–83 months) and that demonstrate adequate psychometric properties across diverse contexts, the first in this age group to the authors' knowledge. Data were harmonized from previous early childhood measurement efforts in 12 countries that all used the same base measurement tool. Data analyses yielded 20 caregiver report items and 84 child direct assessment items (grouped into 16 tasks) that show strong item-level statistics across countries and that cover the domains of early literacy, early numeracy, executive functioning, and social-emotional competencies. Next steps include adding data and items from other measurement tools to the same analytical framework and field testing across a number of contexts and early childhood measurement efforts. The vision is for the resulting core sets of items, along with guidance on data collection, management, and analysis, to serve as global public goods so that they can (i) present a starting point for linking across different early childhood measurement tools for children aged 4–6; (ii) increase quality across measurement efforts; and (iii) facilitate the scale up of early childhood measurement. When supplemented with items that capture local contexts and their measurement needs, these core sets of items should help to advance understanding of universal and context-specific factors that underlie child development and thus help policymakers make decisions that ensure children receive the quality early childhood care and education they need in order to reach their full potential.

## Introduction

Evidence from a range of disciplines confirms that a child's earliest years are a critical time to invest in building human capital. The returns to investments in the early years are diverse and include: improved cognitive skills, reduced repetition and drop-out rates, and development of the social-emotional competencies needed to succeed in the workplace and in adulthood [for example, (1–6)]. In response to the overwhelming evidence of impact, unmet need, and growing demand for early childhood services, governments and international organizations are giving increasing priority to investments during children's early years. This increased prioritization has manifested itself most clearly in improved access to pre-primary education. Global enrollment in pre-primary education has increased dramatically in the last decade from 32 percent in 2000 to more than 50 percent in 2017. In low- and middle-income countries, enrollment in pre-primary education has doubled in the same time period, from 18 to 36 percent. This increasing trend can be seen across all regions of the world and is accelerating (7).

Despite improvements in access, however, in many countries, the quality of early childhood education is low and unlikely to promote significant improvements in children's development. Experience from high-, middle-, and low-income countries alike demonstrates that even when access to early childhood education goes up, children's outcomes do not always improve [for instance, (8–12)]. Without an adequate emphasis on quality, children will not reap potential benefits, parents may be less likely to enroll their children in pre-primary education, and systems will waste resources. The last two decades of experience in the basic education sector [within the context of trying to achieve Universal Primary Education and the current global learning crisis; (13)] provide a preview of what could happen if pre-primary education continues to be scaled without adequate quality.

Within this context, it is critical for governments, development partners, and parents to have information on children's development in the preschool years (here defined as ages 4–6) and to generate data in a way that can foster improvements in programs and policies (14, 15). Governments, and not just academic researchers, should be able to implement measures of child development at scale. This implies the need for relatively straightforward measures accompanied by guidance on implementation protocols. In addition, the resulting data needs to be reliable with validity evidence for recommended uses of the scores. Without this information from these kinds of measures, programs and policies are unlikely to be as effective, efficient, and equitable as they could be, and policymakers risk making decisions with limited information.

There have been various global efforts to promote measurement of children's development in the preschool years. These include the Measuring Early Learning Quality and Outcomes (MELQO) project (16, 17), early Human Capability Index [eHCI; (18)], East Asia-Pacific Early Child Development Scales [EAP-ECDS; (19)], Regional Project on Child Development Indicators [PRIDI; (20)], and the International Development and Early Learning Assessment [IDELA; (21)].There has been an important emphasis within these initiatives on local applicability and relevance, and this should rightly be prioritized to ensure the resulting data are meaningful and useful to governments and local stakeholders. There also have been investigations into the cross-context comparability of the data generated by specific tools [for example, (18, 22)].

However, while there has been this proliferation of child outcomes measurement efforts and data, it has been difficult to get an accurate view of how child development varies across the globe because the measures used do not always capture the same constructs in the same way. While these measures tend to target similar developmental domains (e.g., early literacy, early numeracy, executive functioning, and social-emotional competencies), the items used often differ from measure to measure so that it is not possible to link the resulting data across different measurement efforts. This situation is compounded by the fact that there is no large-scale international measurement program at the pre-primary level in which the same items are administered to representative populations at a regular frequency. This is in contrast to assessment at the primary and secondary levels, which has initiatives like the Programme for International Student Assessment [PISA; (23)], Trends in International Mathematics and Science Study [TIMSS; (24)], and Progress in International Reading Literacy Study [PIRLS; (25)].

In order to link across tools, there needs to be a common set of items embedded in each tool (26). For this to be feasible across a wide range of measurement efforts, the common set of items needs to have three key features. First, the set needs to be short enough so that adding it to an existing tool does not make the overall measurement exercise excessively burdensome (27). Second, the common set of items needs to demonstrate acceptable psychometric properties across as wide a range of contexts as possible in order to ensure the quality of the data collected in each context. These properties would include an average level of difficulty for children in the age range of interest and a consistent scoring pattern with other items measuring the same developmental domain. Third, the core set of items, along with guidance on data collection, management, and analysis, needs to be openly accessible by a wide range of stakeholders, including national statistical offices and researchers.

Identifying these items would allow for increased efficiencies and synergies across data collection efforts, for example through the provision of common guidance around data collection, management, and analysis, thus helping to ensure that national statistical offices and researchers are collecting comparable and relevant data with high-value information on child development in the preschool years. It is crucial to note that these core sets of items can and should be supplemented with items that are more specifically suited to local contexts and needs in order to ensure that the resulting data provide information relevant to local policy and practice (28).

There are currently efforts to identify sets of items that demonstrate adequate psychometric properties across contexts for children aged 24–59 months in the context of the sustainable development goal (SDG) 4.2.1 and for children aged 0–36 months—the Early Childhood Development Index [ECDI2030; (29)] and the Global Scale for Early Development [GSED; (30)], respectively. Preschool systems, however, often focus on children aged 4–6 (ages 48–83 months). The core sets of items described in this paper are thus aimed at children aged 4–6 and are intended to complement the other ongoing global efforts.

The goal of the work presented in this paper was to identify core sets of caregiver-report items and direct assessment items that measure key developmental domains for children aged 4–6 (48–83 months) and demonstrate adequate psychometric properties across multiple contexts. Data from previous early childhood measurement efforts using the same base assessment tools in 12 countries were harmonized and analyzed. This work represents a step toward the generation of psychometrically robust core sets of items that can be made global public goods so that they can be embedded into measures of early childhood development outcomes. The methods, results, and subsequent discussion are presented below.

## Methods

### Research Design and Participants

The data for this study were obtained from previous data collection initiatives over the last 5 years conducted by governments and supported by country and regional teams within the World Bank. These data collection initiatives, done initially for either population monitoring or program evaluation purposes, were carried out independently. However, they all used the same base measurement tools (i.e., a caregiver report and/or a child direct assessment) described below.

As summarized in Table 1, participants in this study were caregivers (*n* = 16,015) and children (*n* = 24,533) from 12 different countries located in Africa (7 countries), Asia (3 countries), Central America (1 country), and South America (1 country). Most of the children in this study were aged between 48 and 72-months, although data from younger and older children were included in the estimation of the items' psychometric properties described in the following sections.

**Table 1:** Total sample size and sample size by children's age by country.

**Country (Year)**	**Caregiver report**					**Direct assessment**				
	**Total *n***	** <48 mos**	**48–60 mos**	**60–72 mos**	**>72 mos**	**Total *n***	** <48 mos**	**48–60 mos**	**60–72 mos**	**>72 mos**
Ethiopia (2017)	**212**	14	57	124	17	**1,144**	30	150	399	565
Kenya (2017)						**2,647**	521	1,227	683	216
Laos (2016)	**9,353**	1,150	1,929	2,286	2,146	**9,353**	1,150	1,929	2,286	2,146
Lesotho (2017)	**985**	4	192	529	135	**985**	4	192	529	135
Madagascar (2016)	**200**	35	77	63	25					
Mongolia (2015)						**2,959**		926	1,914	101
Nigeria (2019)	**160**	1	16	42	23	**160**	1	16	43	23
Pakistan (2018)	**672**	206	254	144	41	**672**	206	254	144	41
Sudan (2015)						**166**	1	4	56	25
Tanzania (2017)						**1,165**	22	64	214	650
Central American country (2016)	**696**	134	221	265	65	**814**	356	265	65	0
South American country (2017)	**3,737**	0	30	1,479	2,218	**4,468**	0	1,911	2,552	4
Total	**16,015**	**1,544**	**2,776**	**4,932**	**4,670**	**24,533**	2,291	6,938	8,885	3,906

*“n” refers to sample size. Any discrepancy of sample size by age and the total sample size is due to missing data on children's age for some participants. Empty rows in a country indicate that the country team did not administer the measurement tool as part of their project. The Central and South American countries shared their data on condition of anonymity*.

### Materials

Country and regional teams had administered assessments of early childhood development using a caregiver report questionnaire and/or a child direct assessment drawn from the MELQO suite of tools (17). These tools were designed to generate data on early child development and the quality of early learning environments in low- and middle-income countries. The caregiver report questionnaire and child direct assessment capture aspects of child development across four domains: early literacy, early numeracy, executive functioning, and social-emotional competencies.

The total number of items included in these measurement tools varied by country as country teams added or removed items depending on their project objectives, local context, or at the request of governments. Once all items were pooled, there were a total of 92 items that had been administered as part of the caregiver report questionnaire and 146 items that had been administered as part of the child direct assessment. Table 2 shows the breakdown of these items for each tool, by developmental domain. All identified items were analyzed in this study.

**Table 2:** Number of initial items by developmental domain.

**Caregiver report questionnaire**		**Child direct assessment**	
Early literacy	17 items	Early literacy	50 items
Early numeracy	24 items	Early numeracy	42 items
Executive functioning and Social-emotional competencies	41 items	Executive functioning	46 items
		Social-emotional competencies	8 items

#### Caregiver Report Questionnaire

This questionnaire is administered to the child's primary caregiver at home (16, 17). Trained enumerators present the standardized questions to caregivers who report whether their child exhibits specific behaviors; caregivers can also respond that they do not know the answer to a particular question. Additional sociodemographic information about the caregiver, the child, and their home is collected during the administration of this questionnaire.

The early literacy domain section of the questionnaire includes items related to the child's alphabet knowledge, expressive vocabulary, and listening comprehension. The early numeracy items questionnaire includes items focused on verbal counting, set production, mental addition, numeral identification, spatial sense, and measurement vocabulary. Finally, the items measuring executive functioning and social-emotional competencies include questions about the child's self-regulation, social cognition, social competence, and emotional well-being.

#### Child Direct Assessment

The direct assessment is focused on exploring what the child knows and can do by asking the child to perform various tasks (16, 17). Trained enumerators administer each item to the child in a one-to-one interaction. The enumerator marks whether the child can or cannot perform a specific task and may also indicate whether particular items were not administered to the child for unexpected reasons. Key demographic information (e.g., age and gender) about the child are also captured during the administration of the assessment.

The early literacy section of the assessment includes items related to the child's alphabet knowledge, phonological awareness, expressive vocabulary, and listening comprehension. The early numeracy section includes items focused on verbal counting, set production, mental addition, numeral identification, spatial sense, and measurement vocabulary. Finally, the section measuring executive functioning and social-emotional competencies includes items that capture the child's working memory, inhibitory control, social cognition, and emotional self-knowledge.

### Procedure

The caregiver report questionnaire and child direct assessment were adapted to the countries' local contexts before implementation; as a result, there were variations in how some of the items were worded, scored, and labeled. Thus, before data analysis, the data first had to be harmonized across datasets. This involved mapping items across implementations to identify (i) which items were implemented in the same way across contexts, (ii) which items were adapted slightly but were still equivalent across implementations, (iii) which items were adapted so that they were not equivalent across implementations, (iv) which items were completely new, and (v) which items from the original tools were omitted in a given implementation. This was followed by the systematic recoding of data sets using a standard data codebook for all items, which resulted in uniform scores and labels across data sets. The authors can be contacted for more information about the harmonization process, which has been documented and is available upon request.

After the item-level data were harmonized, experts in early childhood development reviewed the items for face and content validity to ensure that the measurement tools adequately represented the developmental domains they intended to measure (31, 32). Psychometric analyses employing both Classical Test Theory (CTT) and Item Response Theory (IRT) frameworks were used to identify items with the psychometric characteristics described below. Invariance analyses were not conducted as part of this study since the authors' main objective was to first identify items that demonstrated consistently robust psychometric properties for children aged 4–6 within each country. We expect to conduct additional studies in the future that explore the invariance properties and differential item functioning of the core set of items for different groups and contexts.

In most cases, the items in the caregiver report and child direct assessment tools were scored only as either an incorrect answer (0 = “*cannot do it yet*”) or a correct answer (1 = “*can do it*”). As mentioned above, country teams were allowed to adapt the measurement tools to the local context. Therefore, some versions of the caregiver report questionnaire included items scored as either an incorrect answer (0 = “*cannot do it yet*”), a partially correct answer (1 = “*can do it with help*”), or a correct answer (2 = “*can do it independently*”). Thus, the analyses distinguished between statistics for dichotomous items and ordinal items.

The main objective of the analysis was to identify items with high-information content representing multiple domains of child development that can be used across countries and that capture the progress that tends to happen over time as children develop normally. The item-level statistics calculated included measures of item difficulty, item discrimination, item impact on internal consistency, item factor loadings, and item relationship with age controlling for other covariates. Table 3 summarizes each of the statistics estimated in order to identify items that show robust psychometric properties across countries (31–36).

**Table 3:** Item-level statistics and inclusion criteria for selected items.

**Item statistic**	**Inclusion criteria for dichotomous items**	**Inclusion criteria for ordinal items**
**CTT difficulty**. The proportion of examinees answering the item correctly	Items with difficulty between 0.10 and 0.90 were included	Items with <0.90 of the cases scoring on either the highest or lowest answer options were included
**CTT discrimination**. It refers to the item's capacity to distinguish examinees with high and low ability based on their total score	Item-domain correlation above 0.30 Item-domain correlation with item excluded from domain total score above 0.10 Item-total test score correlation above 0.25	Item-domain correlation above 0.30 Item-domain correlation with item excluded from domain total score above 0.10 Item-total test score correlation above 0.25
**Item contribution to internal consistency**. Each item contributes to increase or decrease the internal consistency depending on its amount of covariance with other items measuring a common developmental domain	Items that increase Cronbach's Alpha coefficient when included as part of the developmental domain	Items that increase Cronbach's Alpha coefficient when included as part of the developmental domain
**Developmental domain internal structure**. Items should be associated with the domain they intend to measure. CFA techniques empirically determined this relationship	Items with standardized factor loadings above 0.40 Items with positive standardized factor loadings below 0.40 were reviewed by experts	Items with standardized factor loadings above 0.40 Items with positive standardized factor loadings below 0.40 were reviewed by experts
**Relationship with age**. Given its association with psychological development, age could be considered an external criterion to identify items that intend to measure development	Categorical regression models that predict the item response based on age and sociodemographic covariates (e.g., gender, preschool enrollment status, mother's education level, if available) Items with a positive regression coefficient for age	Ordinal regression models that predict the item response based on age and sociodemographic covariates (e.g., gender, preschool enrollment status, mother's education level, if available) Items with a positive regression coefficient for age
**IRT estimates**. These additional item level statistics inform about optimal difficulty or discrimination	1PL and 2PL IRT item difficulty included between −3 and 3 2PL item discrimination above 0.50	GRM item discrimination above 0.50

*“1PL” and “2PL” refer to the Rasch and two-parameter logistic IRT models. “CFA” refers to Confirmatory Factor Analysis*.

These statistics were estimated for each country-dataset separately. After the content and psychometric analyses within each country, items were classified using four levels:

- Tier 1: Items met all the criteria described above.
- Tier 2: Items that failed to meet just one criterion. These items were included in a second round of psychometric analyses since some CTT statistics tend to improve when other inadequate items are removed.
- Tier 3: Items that did not meet two or three criteria. In these cases, item content was reviewed by experts in developmental psychology to determine whether they should be part of a core set of items.
- Tier 4: Items that did not meet more than three criteria. These items were excluded from any subsequent analyses.

Items in Tiers 1, 2, and 3 were included in a second round of within-country psychometric analyses using the same statistics and measurement models used in the first round (Table 3). This second round of analyses is particularly important for some CTT correlations and factor analysis estimates, which are prone to change when items are dropped (31, 32). All remaining items were tiered once again within countries after this second round of psychometric analyses.

Next, the proportion of correct responses by age was calculated for each of the remaining items to identify those with medium difficulty in the age range of interest for the study. Items were defined to have medium difficulty either when between 40 and 60 percent of the caregivers indicated that their child was able to perform a specific task or exhibited a particular behavior, or when the same proportion of assessed children were able to perform a specific task correctly. At this stage, only items that had been applied in at least two countries were retained in the core.

Annex A includes Tables A1 and A2, which summarize the tier placement of each item from the Caregiver Report Questionnaire and Child Direct Assessment, both at the country level and across countries. As summarized in these tables, in most instances, items included as part of the core set were identified in tier 1 in the within-country analyses. To a lesser extent, items were identified in tiers 2 or 3. Only rarely were items identified in tier 4. In the infrequent case that an item produced suboptimal statistics that placed it in tier 4 in one country, but in higher tiers in other countries, a content review by experts in developmental psychology was carried out to determine if the item should be included or not.

Annex A also includes Tables A3 and A4, which present the psychometric results for each item. In the interest of brevity, country-level psychometric results for each item are summarized in terms of average estimates and standard deviations across countries. For each statistic reported in these two tables, the average estimate is simply the arithmetic mean calculated by adding up country-level estimates and dividing this sum by the number of countries where the item was included. The standard deviation is calculated by calculating the sum of squares between this arithmetic mean and each country-level estimate, dividing it by the number of countries where the item was included minus one and obtaining the square root of this ratio.

For all analyses described here, observations with missing information were excluded from analyses (that is, missing responses were not recoded as incorrect responses) and multiple imputation procedures were not carried out to estimate plausible values at the item level.

## Results

Following the analytical process described in the previous section, a core set of items were identified. These items represent adequate content coverage as defined by experts in early childhood development, robust psychometric properties across countries, and empirical evidence of medium difficulty for children aged 4–6 years old. The results section summarizes the psychometric properties of the core set of items in the caregiver report questionnaire and the child direct assessment protocol.

### Caregiver Report Questionnaire

Data from eight different countries –Ethiopia, Laos, Lesotho, Madagascar, Nigeria, Pakistan, and two Central and South American countries that shared data on the condition of anonymity—were used to identify a robust core set of items from the caregiver report questionnaire. After the psychometric analyses were performed, 69 out of the 92 unique original items were placed in tiers 1–3 given their satisfactory psychometric properties within countries; however, only 20 of these items showed medium difficulty for children aged between 4 and 6-years-old both within countries and across countries. Of these 20 core items, five correspond to the early literacy developmental domain, six to early numeracy, and nine measure executive functioning and social-emotional competencies.

Table 4 lists the caregiver report core items and their tier level. Overall, early literacy and early numeracy core items tended to show better psychometric properties compared to the executive functioning and social-emotional competencies core items.

**Table 4:** List of core items from the caregiver report questionnaire.

**Domain**	**Item**	**Tier**	**Number of countries**
Literacy	1. Names at least 10 letters	2	5
Literacy	2. Reads four simple words	2	3
Literacy	3. Reads/follows the text in a correct direction from left to right and from top to bottom? (even if they cannot read)	1	4
Literacy	4. Writes at least three letters or some letters in his/her name	1	4
Literacy	5. Writes a simple word	1	4
Numeracy	6. Can count from 1 to 10	3	4
Numeracy	7. Can count from 1 to 20	1	4
Numeracy	8. Knows the difference between tall and short using two animal examples.	2	6
Numeracy	9. Knows the difference between heavy and light using two animal examples.	3	6
Numeracy	10. Can tell if it is yesterday, today, or tomorrow	1	4
Numeracy	11. Knows that a one-digit number is more than another one-digit number (e.g., 4 is more than 2)	1	4
EF&SE	12. Pays attention when doing an activity	2	2
EF&SE	13. When asked to do several things, remembers all the instructions	2	6
EF&SE	14. S/he is able to plan ahead	2	5
EF&SE	15. Stops an activity when told to do so	3	7
EF&SE	16. Keeps working at something until s/he is finished	2	7
EF&SE	17. Gets along with other children s/he plays with	3	6
EF&SE	18. Adjusts easily to transitions (for example, to a new teacher or classroom)	2	5
EF&SE	19. Accepts responsibility for his/her actions	3	7
EF&SE	20. Settles down after periods of exciting activity	3	6

*“Literacy” refers to the Early Literacy developmental domain, “Numeracy” to the Early Numeracy developmental domain, “EF&SE” to the Executive Functioning & Social-emotional Competencies development domain. “Number of countries” refers to the number of countries in which the item was included in the caregiver report*.

Table A3 in Annex A presents a list of the core items in the Caregiver Report Questionnaire along with their average and standard deviation item-level statistics. These statistics aggregate item-level estimates from countries in which the item was included in the questionnaire. In terms of average CTT difficulty (see column 2 in Table A3), it ranged from an average easy item in the case of the early numeracy item “*Can count from 1 to 10*” (mean = 0.85, s.d. = 0.16) to a moderately difficult literacy item “*Writes a simple word*” (mean = 0.34, s.d. = 0.14). Note that these CTT average difficulty statistics were calculated using data from each country separately, then pooled together as the summary statistics included in Table A3. The CTT difficulty estimates from each country were calculated with data from children of different ages ranging from 2 up to 10 years old; still, the final core items tended to show average difficulty for children aged 4–6 years old within each country. Moreover, no item showed ceiling or floor effects (see columns 1 and 2 in Table A3).

All items show positive average correlations both with the domain and the total score, indicating a good level of CTT discrimination and item contribution to internal consistency (see columns 3–5 in Table A3). The standardized factor loadings for each core item and their corresponding latent developmental domain are positive and above 0.30, which indicates that the items are strongly related to the construct they intend to measure (see column 6 in Table A3).

In column 7 in Table A3, all average values are positive except for two items: the cognitive and social-emotional competencies development items “*Pays attention when doing an activity*” and “*Gets along with other children s/he plays with*.” These negative coefficients indicate that as children get older, caregivers are less likely to respond that the child exhibits these two behaviors; however, these items were retained given that they presented adequate levels of difficulty and discrimination. For the rest of the core items, the positive average coefficients imply that older children are more likely to show a specific behavior or correctly execute a particular task according to the information gathered from their caregivers.

Finally, the core items show adequate to high discrimination, indicating that items can distinguish between respondents at lower and higher levels of development (see column 8 in Table A3). The average IRT difficulty values from the 1-PL (one-parameter logistic or Rasch model) and 2-PL (two-parameter logistic model) models are within the recommended interval and imply that items were neither extremely easy nor extremely difficult (see columns 9 and 11 in Table A3). The two IRT models consistently indicate that the easiest item is “*Can count from 1 to 10*,” while the most difficult is “*Writes a simple word*.”

### Child Direct Assessment

Child assessment data from ten different countries –Ethiopia, Kenya, Laos, Lesotho, Nigeria, Pakistan, Sudan, Tanzania, and two Central and South American countries that shared data on condition of anonymity—were used to identify a robust core set of items for direct assessment. Of the 146 unique original items, 120 items showed adequate psychometric properties within countries, but only 84 items showed medium difficulty for children aged 4–6 years old both within countries and across countries. Of those 84 core items, 27 items measure early literacy, 29 early numeracy, 27 executive functioning, and only one item measures the social-emotional competencies domain.

Several of the core items in the direct assessment tool are part of larger tasks that have more items; in most cases, all or almost all of the items in a task were found to be psychometrically robust. Table 5 lists all core items and tasks in the child direct assessment protocol by tier level.

**Table 5:** List of core items and tasks from the child direct assessment protocol.

**Domain**	**Task**	**Tier level**				**Number of countries**
		**1**	**2**	**3**	**Total items**	
Literacy	1. Letter identification task	14	3	0	17	10
Literacy	2. Listening comprehension task	0	0	4	4	10
Literacy	3. Initial sound discrimination tasks	2	1	0	3	7
Literacy	4. Letter sound identification tasks	0	1	1	2	2
Literacy	5. Writing names	0	1	0	1	7
Numeracy	6. Number comparison task	0	1	0	1	8
Numeracy	7. Number identification task	10	0	0	10	10
Numeracy	8. Producing a set task	3	0	0	3	10
Numeracy	9. Simple addition and subtraction task	5	0	0	5	10
Numeracy	10. Mental transformation task	0	1	2	3	6
Numeracy	11. Naming shapes task	1	2	0	3	4
Numeracy	12. Object spatial position identification task	2	1	1	4	10
Executive Functioning	13. Head Toes Knees Shoulders task	2	9	1	12	8
Executive Functioning	14. Pencil tap task	12	0	0	12	3
Executive Functioning	15. Forward and backward digit span task	2	1	0	3	10
Social-emotional	16. Emotion identification task	1	0	0	1	7
	**Total items**	**56**	**22**	**9**	**84**	

*“Literacy” refers to the Early Literacy developmental domain, “Numeracy” to the Early Numeracy developmental domain, “EF” to the Executive Functioning Developmental Domain, and “Social-emotional” to the Social-emotional Competencies development domain. “Number of countries” refers to the number of countries in which the task was included in the child direct assessment*.

In the case of the early literacy domain, core tasks focused on letter identification, oral story comprehension, initial sound discrimination, letter sound identification, and the child's ability to write their name.

For the early numeracy domain, one item focused on the comparison of one-digit numbers. Additional early numeracy core tasks measure number identification, set production, simple addition, mental transformation, naming shapes, and object spatial position identification. For the executive functioning developmental domain, the self-regulation tasks of pencil tap and head-toes-knees-shoulders showed robust psychometric properties, as well as some memory items focused on forward and backward digit span. In the social-emotional competencies domain, only one item measuring emotion identification yielded optimal psychometric properties.

Table A4 in Annex A contains summary item-level statistics for Child Direct Assessment core items. The average CTT difficulty statistics ranged from an easy literacy item “*Listening comprehension task. Q1*” (mean = 0.84, s.d. = 0.11) to a moderately difficult item also part of the early literacy domain “*Initial sound discrimination tasks. Q2*” (mean = 0.28, s.d. = 0.22). As indicated for the Caregiver Report Questionnaire, the CTT average difficulty statistics were calculated using the complete data collected in each country, including children of different ages. The final core items tend to show average difficulty in the case of children aged 4–6 years old. No core item showed ceiling or floor effects (see column 1 in Table A4).

In summary, all core items showed positive correlations with both domain and total scores, which indicates that items are discriminating between low- and high-scoring children and contributing to internal consistency (see columns 2–4 in Table A4). In terms of the standardized factor loadings between the items and their latent developmental domain, results indicate that items are strongly related to the construct they intend to measure. Moreover, all age coefficients are positive, which implies that older children are more likely to respond to the items correctly compared to their younger peers (see column 6 in Table A4).

Consistent with the CTT difficulty results presented above, the 1PL IRT difficulty estimates show that items are neither extremely easy nor difficult (see column 7 in Table A4). However, the results of the 2PL IRT model indicate that two items—“*Initial Sound Discrimination Q1*” and “*Mental transformation Q1*”—might be easier than expected when controlling for item discrimination (see column 9 in Table A4). All items yielded a positive average 2PL IRT discrimination estimate, indicating that they can distinguish between respondents at lower and higher levels of development in the four measured domains (see column 8 in Table A4).

## Discussion

The goal of the work presented in this paper was to identify a core set of caregiver-report items and a core set of direct assessment items that measure key developmental domains for children aged 4–6 and that demonstrate adequate psychometric properties across multiple contexts. This work represents a step toward the generation of psychometrically robust core sets of items that can be made global public goods so that they can be embedded into measures of early childhood development outcomes to enable linking across tools and comparability of data. As indicated in the Introduction, the linking of results across different ECD measures can be achieved when all of the measurement tools concerned share a proportion of common items. If the tools do not share any common items, a separate set of items (such as the core items presented here) could be administered alongside these tools to the same sample of participants, and this would allow the scores from one measure to be expressed in terms of the other. Such linking approaches are commonly used in educational psychology and large-scale learning assessment studies to track learning trends over time or to compare the results from two or more learning assessments measuring the same domain (37, 38).

Moreover, identifying and promoting the use of psychometrically robust core sets of items, along with accompanying guidance, will improve the quality and efficiency of measurement efforts and help policymakers and other stakeholders to collect data that have high information content and relevance for child development in the preschool years.

This study capitalized on the fact that the World Bank supported multiple early measurement efforts across the globe and that many of these measurement efforts used the same base tools (drawn from the MELQO suite of tools) for measuring child outcomes in the preschool years (14, 16, 17). Thus, this presented a unique opportunity to compile and analyze data from 12 countries in order to identify items that demonstrate adequate psychometric properties for the target age range across contexts. The analyses yielded 20 caregiver report items and 87 child direct assessment items (grouped into 17 tasks) that showed strong item-level statistics across countries. These item-level statistics include item difficulty, item discrimination, item contribution to internal consistency, item standardized factor loadings, and item relationship with age controlling for other covariates. These items covered all early child development domains of interest, namely early literacy, early numeracy, executive functioning, and social-emotional competencies.

It is important to emphasize that the core sets of items identified in this paper do not capture all relevant data for all early childhood development measurement purposes. For example, while they might serve some monitoring purposes, they would likely need to be supplemented with additional items in order to provide a more nuanced set of data on early childhood development outcomes for purposes such as impact evaluations. One way of addressing this would be to embed the core sets of items into other commonly used measures of early childhood development. Preliminary comparisons of these core item sets with four other commonly used measures—eHCI (18), EAP-ECDS (19) PRIDI (20), and IDELA (21)—show overlap between the core sets of items and items that comprise these other measures (see Table B1 in Annex B); this item overlap implies that relatively few items will need to be added to these measures to ensure they contain the complete core sets of items. The ECDI2030 (29) and GSED (30) were not included in Table B1 due to differences in the targeted age range, which imply that few items would overlap—this was confirmed by the fact that only two items from the proposed core set of caregiver report items (targeted at children aged 48–83 months) overlap with the ECDI2030 (targeted at children aged 24–59 months).

In addition, there may be a need to supplement the core set of items with additional questions or indicators that more specifically respond to the needs of local contexts in order to ensure that the resulting data provide information relevant to local policy and practice (28). This could be done by determining which locally-relevant constructs the core items do not cover and then identifying items from existing measures that capture these constructs to add on to the core set of items. If items capturing the desired constructs cannot be found, it may also be possible to develop new, contextually-specific items to add on to the core set of items by working with local stakeholders, experts in child development, and psychometricians (39).

There are, however, some limitations to the current analyses and some critical next steps that should be completed before considering the core sets of items identified in this paper as ready to embed in future measurement efforts. First, while the analyses and results indicate a set of items measuring social-emotional competencies that could be included in the core, these items demonstrated poorer psychometric properties than the items measuring early literacy and numeracy. None of the caregiver report items capturing social-emotional competencies were categorized as Tier 1 items, and there was only one item from the child direct assessment that met the desired criteria for inclusion for children aged 4–6 years old. This is consistent with past findings showing that literacy and numeracy items consistently perform better on the psychometric parameters chosen for the current analysis across contexts than do items focused on social-emotional competencies [see, for example, (6, 14, 18, 22, 38, 40–44)], and may be linked to the fact that social-emotional competencies are more contextually and culturally specific. Thus, it is more challenging to find social-emotional items that perform similarly well psychometrically across countries. This result has implications for cross-context comparisons of children's social-emotional development and suggests that further work needs to be done to strengthen this aspect of the core sets of items.

Second, keeping in mind that a key feature of a core set of items is parsimony, the number of items currently included in the core set of child direct assessment items may still be too many to feasibly incorporate into existing measures. With 84 items grouped into 16 tasks, it is unlikely that stakeholders will be able to add many more context-specific items to the core and still be able to administer the resulting assessment to a 4–6 year old within an acceptable period of time. These 84 items would likely take ~30 min to administer, which is the upper bound to what could be reasonably expected of a preschooler. Thus, while these items demonstrate adequate psychometric properties across contexts, they will need to be further trimmed down using additional considerations. Given the need in many contexts to rely on non-expert enumerators to collect these data and the potential for this to introduce measurement error, priority could be given to items that are less complex to administer and that do not exhibit sensitivity to the expertise of the enumerator conducting the interview or assessment. In addition, some tasks in the core tap into the same developmental constructs; thus, the core set of items could be further trimmed by keeping only one task per developmental construct, perhaps prioritizing items that already overlap with other measurement tools. Finally, further analyses could be run to try to identify a subset of the items that provide roughly the same amount of information as the full set.

On the other hand, the 20 core caregiver report items represent a manageable number of items that could be incorporated into existing measures of child outcomes. This core set of items also lends itself to inclusion in household surveys as a means of scaling up the measurement of early child development at the population level and enabling child outcome data to be linked to contextual information commonly collected via household surveys such as household characteristics, access to health services and social protection programs, and consumption data. It is worth noting here that the ECDI2030 (29) was designed for implementation through household surveys (particularly UNICEF's Multiple Indicators Cluster Survey), and it too contains 20 items.

Third, the analyses presented here all drew from the same base measurement tools from the MELQO (16, 17). A critical next step would be to expand the harmonized dataset to include data and items from other existing measures of child development in the preschool years, in order to ensure that these findings are not specific to the MELQO suite of tools. This would require the strengthening of harmonization protocols and guidelines across measures; the ex-post harmonization protocol developed as part of this work provides a strong base for this effort and represents a step toward a global database of early childhood outcomes in the preschool years. As mentioned above, the fact that there is overlap between the core sets of items and items that currently comprise the commonly used measures of early childhood development also facilitates the expansion of this work to include other measures of child development. It is also worth mentioning here that some items were excluded from the core because they had not been implemented in at least two countries in the current dataset; by drawing on additional data, it may be possible to re-evaluate whether these items should also be considered for inclusion in the core.

Another critical step would be the field-testing of the identified items as a group in different measurement and country contexts. In the current analysis, all items did not appear across all of the countries in the sample and field protocols were slightly different in each country. Moreover, the objective of measurement also varied (sometimes it was population monitoring, sometimes it was program evaluation), which would have influenced the profile of enumerators and the intensity of quality assurance in data collection. It will be important to check under which conditions the items retain their psychometric properties. Standardizing items and field protocols will also permit an exploration of whether these items function similarly and an evaluation of measurement invariance across contexts.

This field-testing would also reveal the extent to which the caregiver report and direct assessment data from the cores corroborate each other when implemented together in the field. It is important to flag here, however, that while the core set of caregiver-report items and the core set of direct assessment items capture the same key developmental domains, the items in the caregiver report and the direct assessment cores do not correspond directly (i.e., the items do not target exactly the same skills or competencies within each domain). Nevertheless, a comparison of the two measures could provide convergent validity for the use of the two types of tools.

Finally, it would be important to explore the predictive validity of these items. At this point in time, there are no standardized longitudinal data that could be drawn on to feed into the selection of the core sets of items. It is worth noting, however, that predictive validity was one of the criteria used when items were originally selected for inclusion in the MELQO (17).

This paper presents the work done to identify two core sets of caregiver-report items and direct assessment items that measure key developmental domains for children aged 4–6 and that demonstrate adequate psychometric properties across multiple contexts. They represent a starting point for (i) linking across different early childhood measurement tools for children aged 4–6; (ii) increasing quality across measurement efforts; and (iii) facilitating the scale up of early childhood measurement. This would be hugely beneficial to the field of child development as a whole, as it would help to advance understanding of universal and context-specific factors that underlie child development and thus help policymakers make decisions that ensure children receive quality early childhood care and education they need in order to reach their full potential.

## Data Availability Statement

The data analyzed in this study is subject to the following licenses/restrictions: These data need individual government approval for access. Requests to access these datasets should be directed to Adelle Pushparatnam, apushparatnam@worldbank.org.

## Ethics Statement

Ethical review and approval was not required for the study on human participants in accordance with the local legislation and institutional requirements. Written informed consent from the participants or the participants' legal guardians was not required to participate in this study in accordance with the national legislation and the institutional requirements.

## Author Contributions

AP led the writing of the article and contributed to the data analysis and interpretation of the results. DL led the data analysis and interpretation of the results. AH and MC contributed to data harmonization efforts, led by JA. AD led the World Bank's early childhood country engagements and early childhood measurement efforts from 2014–2018. All authors contributed to the article and approved the submitted version.

## Conflict of Interest

The authors declare that the research was conducted in the absence of any commercial or financial relationships that could be construed as a potential conflict of interest.
