# Supplementary material for: Measuring Early Childhood Development Among 4–6 Year Olds: The Identification of Psychometrically Robust Items Across Diverse Contexts
Source: Front Public Health. 2021 Feb 3;9:569448. doi: 10.3389/fpubh.2021.569448 (PMC7888256; doi:10.3389/fpubh.2021.569448)
Supplement: Supplementary file 1 [file Data_Sheet_1.PDF]

Table A1. ***Items by final tier level and within-country tier level in Caregiver Report Questionnaire.***

Domain	Item	Final tier	Number of countries in which item was placed as in tier				Total number of countries
			1	2	3	4	
**Literacy**	1. Names at least ten letters.	2	3	1	1	0	5
**Literacy**	2. Reads four simple words.	2	2	0	1	0	3
**Literacy**	3. Reads/follows the text in a correct direction from left to right and from top to bottom? (even if they cannot read)	1	4	0	0	0	4
**Literacy**	4. Writes at least three letters or some letters in his/her name	1	4	0	0	0	4
**Literacy**	5. Writes a simple word	1	3	1	0	0	4
**Numeracy**	6. Can count from 1 to 10	3	1	0	3	0	4
**Numeracy**	7. Can count from 1 to 20	1	4	0	0	0	4
**Numeracy**	8. Knows the difference between tall and short using two animal examples.	2	3	2	1	0	6
**Numeracy**	9. Knows the difference between heavy and light using two animal examples.	3	4	0	1	1	6
**Numeracy**	10. Can tell if it is yesterday, today, or tomorrow	1	3	1	0	0	4
**Numeracy**	11. Knows that a one-digit number is more than another one-digit number (e.g., 4 is more than 2).	1	4	0	0	0	4
**C&SE**	12. Pays attention when doing an activity.	2	1	1	0	0	2
**C&SE**	13. When asked to do several things, remembers all the instructions.	2	3	0	3	0	6
**C&SE**	14. S/he is able to plan ahead.	2	1	3	1	0	5
**C&SE**	15. Stops an activity when told to do so.	3	2	2	3	0	7
**C&SE**	16. Keeps working at something until s/he is finished.	2	2	1	4	0	7
**C&SE**	17. Gets along with other children s/he plays with.	3	0	2	3	1	6
**C&SE**	18. Adjusts easily to transitions (for example, to a new teacher or classroom).	2	2	1	2	0	5
**C&SE**	19. Accepts responsibility for his/her actions.	3	3	2	1	1	7
**C&SE**	20. Settles down after periods of exciting activity.	3	1	1	4	0	6

Note: "C&SE" refers to the Cognitive & Social-emotional Competencies development domain.

Table A2. ***Items by final tier level and within-country tier level in Child Direct Assessment.***

Domain	Task and Item.	Final tier	Number of countries in which item was placed as in tier				Total number of countries
			1	2	3	4	
**Literacy 1**	Letter identification; Letter1	1	7	1	1	0	9
**Literacy 2**	Letter identification; Letter2	1	6	0	2	0	8
**Literacy 3**	Letter identification; Letter3	1	8	2	0	0	10
**Literacy 4**	Letter identification; Letter4	1	7	1	1	0	9
**Literacy 5**	Letter identification; Letter5	1	8	1	1	0	10
**Literacy 6**	Letter identification; Letter6	1	7	2	1	0	10
**Literacy 7**	Letter identification; Letter7	1	8	2	0	0	10
**Literacy 8**	Letter identification; Letter8	1	4	1	1	0	6
**Literacy 9**	Letter identification; Letter9	1	7	2	1	0	10
**Literacy 10**	Letter identification; Letter10	2	2	2	1	0	5
**Literacy 11**	Letter identification; Letter11	1	9	0	0	1	10
**Literacy 12**	Letter identification; Letter12	1	7	1	1	0	9
**Literacy 13**	Letter identification; Letter13	1	8	1	1	0	10
**Literacy 14**	Letter identification; Letter14	2	8	1	1	0	10
**Literacy 15**	Letter identification; Letter15	1	7	1	0	0	8
**Literacy 16**	Letter identification; Letter16	2	5	1	1	0	7
**Literacy 17**	Letter identification; Letter17	1	6	0	0	0	6
**Literacy 18**	Listening comprehension; Q1	3	1	3	4	2	10
**Literacy 19**	Listening comprehension; Q2	3	3	3	2	2	10
**Literacy 20**	Listening comprehension; Q3	3	2	3	5	0	10
**Literacy 21**	Listening comprehension; Q4	3	1	2	6	1	10
**Literacy 22**	Initial sound discrimination; Q1	3	3	1	0	2	6
**Literacy 23**	Initial sound discrimination; Q2	2	1	4	0	1	6
**Literacy 24**	Initial sound discrimination; Q3	2	1	1	0	0	2
**Literacy 25**	Letter sound identification; Q1	3	0	1	0	1	2
**Literacy 26**	Letter sound identification; Q2	2	0	2	0	0	2
**Literacy 27**	Name writing	2	5	0	1	1	7
**Numeracy 28**	Number Comparison	2	6	0	1	1	8
**Numeracy 29**	Number Identification, Q1	1	8	1	1	0	10

This is a provisional file, not the final typeset article

**Numeracy 30**	Number Identification, Q2	1	6	3	1	0	10
**Numeracy 31**	Number Identification, Q3	1	6	1	3	0	10
**Numeracy 32**	Number Identification, Q4	1	9	0	1	0	10
**Numeracy 33**	Number Identification, Q5	1	9	0	1	0	10
**Numeracy 34**	Number Identification, Q6	1	7	0	2	0	9
**Numeracy 35**	Number Identification, Q7	1	8	0	2	0	10
**Numeracy 36**	Number Identification, Q8	1	8	1	0	0	9
**Numeracy 37**	Number Identification, Q9	1	7	1	0	0	8
**Numeracy 38**	Number Identification, Q10	1	8	1	0	0	9
**Numeracy 39**	Producing a set, Q1	1	7	2	0	0	9
**Numeracy 40**	Producing a set, Q2	1	2	0	0	0	2
**Numeracy 41**	Producing a set, Q3	1	10	0	0	0	10
**Numeracy 42**	Simple addition, Q1	1	5	0	0	0	5
**Numeracy 43**	Simple addition, Q2	1	10	0	0	0	10
**Numeracy 44**	Simple addition, Q3	1	4	0	0	0	4
**Numeracy 45**	Simple addition, Q4	1	3	0	0	0	3
**Numeracy 46**	Simple subtraction	1	1	1	0	0	2
**Numeracy 47**	Mental transformation; Q1	3	2	1	2	1	6
**Numeracy 48**	Mental transformation; Q2	3	1	0	4	1	6
**Numeracy 49**	Mental transformation; Q3	2	2	4	0	0	6
**Numeracy 50**	Naming shapes Q1	2	2	1	0	1	4
**Numeracy 51**	Naming shapes Q2	2	3	1	0	0	4
**Numeracy 52**	Naming shapes Q3	1	4	0	0	0	4
**Numeracy 53**	Object spatial position identification: Q1	3	7	1	1	1	10
**Numeracy 54**	Object spatial position identification: Q2	1	6	1	2	1	10
**Numeracy 55**	Object spatial position identification: Q3	2	7	1	1	1	10
**Numeracy 56**	Object spatial position identification: Q4	1	6	1	0	0	7
**EF 57**	Head, Shoulders, Knees, Toes, Q1	2	5	1	0	1	7
**EF 58**	Head, Shoulders, Knees, Toes, Q2	1	6	0	1	0	7
**EF 59**	Head, Shoulders, Knees, Toes, Q3	2	4	3	0	0	7
**EF 60**	Head, Shoulders, Knees, Toes, Q4	2	4	2	1	0	7
**EF 61**	Head, Shoulders, Knees, Toes, Q5	2	4	2	1	0	7

**EF 62**	Head, Shoulders, Knees, Toes, Q6	2	6	1	0	0	7
**EF 63**	Head, Shoulders, Knees, Toes, Q7	2	6	1	0	1	8
**EF 64**	Head, Shoulders, Knees, Toes, Q8	2	5	1	0	1	7
**EF 65**	Head, Shoulders, Knees, Toes, Q9	2	6	0	0	1	7
**EF 66**	Head, Shoulders, Knees, Toes, Q10	2	6	0	0	1	7
**EF 67**	Head, Shoulders, Knees, Toes, Q11	3	2	4	0	1	7
**EF 68**	Head, Shoulders, Knees, Toes, Q12	1	4	2	1	0	7
**EF 69**	Pencil Tap, Q1	1	3	0	0	0	3
**EF 70**	Pencil Tap, Q2	1	3	0	0	0	3
**EF 71**	Pencil Tap, Q3	1	3	0	0	0	3
**EF 72**	Pencil Tap, Q4	1	3	0	0	0	3
**EF 73**	Pencil Tap, Q5	1	3	0	0	0	3
**EF 74**	Pencil Tap, Q6	1	3	0	0	0	3
**EF 75**	Pencil Tap, Q7	1	3	0	0	0	3
**EF 76**	Pencil Tap, Q8	1	3	0	0	0	3
**EF 77**	Pencil Tap, Q9	1	3	0	0	0	3
**EF 78**	Pencil Tap, Q10	1	3	0	0	0	3
**EF 79**	Pencil Tap, Q11	1	2	1	0	0	3
**EF 80**	Pencil Tap, Q12	1	2	0	1	0	3
**EF 81**	Backward Digit Span (3 items)	3	4	1	1	2	8
**EF 82**	Forward Digit Span (4 items)	1	6	4	0	0	10
**EF 83**	Forward Digit Span (5 items)	1	8	1	1	0	10
**SE 84**	Identifying emotion	1	4	2	1	0	7

Note: "EF" refers to the Executive Functioning Developmental Domain and "SE" refers to the Social-emotional Competencies development domain.

This is a provisional file, not the final typeset article

Table A3. ***Average item-level statistics for core items in Caregiver Report Questionnaire.***

Item	1	2	3	4	5	6	7	8	9	10	11
**Literacy 1**	.51 (.09)	.43 (.30)	.83 (.12)	.47 (.17)	.36 (.41)	.68 (.14)	.43 (.91)	8.00 (1.76)	-.51 (1.62)	2.15 (.92)	-.13 (.79)
**Literacy 2**		.77 (.21)	.79 (.09)	.43 (.16)	.47 (.16)	.69 (.13)	.04 (.02)		-2.14 (1.57)	2.06 (.97)	-1.35 (1.25)
**Literacy 3**		.55 (.18)	.80 (.08)	.49 (.08)	.53 (.12)	.73 (.07)	.09 (.07)		-.28 (1.31)	1.57 (.37)	-.23 (.65)
**Literacy 4**		.68 (.17)	.87 (.10)	.55 (.10)	.59 (.11)	.84 (.08)	.08 (.04)		-1.23 (1.15)	2.78 (.92)	-.62 (.57)
**Literacy 5**		.34 (.14)	.98 (.02)	.67 (.02)	.61 (.06)	.88 (.08)	.09 (.06)		1.26 (1.41)	4.91 (.88)	.47 (.44)
**Numeracy 6**		.85 (.16)	.66 (.46)	.41 (.17)	.45 (.20)	.74 (.20)	.07 (.02)		-2.79 (1.38)	1.93 (.55)	-1.74 (.98)
**Numeracy 7**		.56 (.14)	.84 (.12)	.43 (.10)	.54 (.14)	.78 (.13)	.07 (.05)		-.38 (.87)	1.72 (.48)	-.28 (.53)
**Numeracy 8**	.07 (.04)	.82 (.14)	.66 (.31)	.36 (.16)	.49 (.19)	.55 (.14)	.68 (1.02)	1.25 (.17)	-2.43 (1.64)	1.82 (.25)	-1.18 (1.15)
**Numeracy 9**	.28 (.03)	.72 (.20)	.66 (.28)	.31 (.18)	.40 (.12)	.52 (.18)	.53 (1.17)	.69 (.47)	-2.13 (1.52)	1.60 (.58)	-1.26 (1.48)
**Numeracy 10**		.78 (.15)	.83 (.15)	.38 (.10)	.42 (.11)	.61 (.20)	.07 (.05)		-2.01 (1.13)	1.70 (.73)	-1.19 (.68)
**Numeracy 11**		.69 (.22)	.90 (.10)	.56 (.12)	.58 (.11)	.83 (.15)	.06 (.05)		-1.29 (1.56)	5.99 (6.08)	-.65 (.66)
**C&SE 12**	.10 (.03)	.50 (.05)	.61 (.12)	.48 (.15)	.49 (.11)	.62 (.14)	-.05 (.37)	1.89 (1.05)			.12 (.00)
**C&SE 13**	.06 (.03)	.46 (.08)	.44 (.23)	.33 (.12)	.40 (.17)	.39 (.16)	.12 (.28)	1.06 (.31)	.78 (.00)	.87 (.00)	.33 (.43)
**C&SE 14**	.28 (.11)	.34 (.10)	.51 (.16)	.34 (.07)	.45 (.17)	.38 (.18)	2.19 (1.83)	.70 (.09)	.17 (.00)	1.28 (.00)	.26 (.11)
**C&SE 15**	.10 (.06)	.43 (.07)	.46 (.12)	.27 (.09)	.31 (.20)	.33 (.20)	.30 (.60)	.77 (.20)	.96 (.00)	.98 (.00)	.33 (.40)
**C&SE 16**	.09 (.08)	.51 (.15)	.52 (.13)	.37 (.11)	.40 (.17)	.42 (.25)	.40 (1.87)	1.42 (.51)	-.01 (.00)	1.05 (.00)	.09 (.10)
**C&SE 17**	.03 (.02)	.76 (.12)	.45 (.19)	.28 (.12)	.34 (.24)	.32 (.31)	-.69 (1.06)	1.16 (.50)	-1.37 (.00)	1.36 (.00)	-.33 (.64)
**C&SE 18**	.14 (.04)	.49 (.19)	.43 (.20)	.31 (.11)	.38 (.23)	.35 (.24)	1.01 (1.35)	.67 (.34)	-.84 (.00)	1.10 (.00)	-.31 (.63)
**C&SE 19**	.14 (.08)	.53 (.11)	.53 (.18)	.37 (.14)	.43 (.20)	.47 (.28)	.52 (.98)	1.12 (.83)	-.35 (.00)	1.17 (.00)	.06 (.25)
**C&SE 20**	.16 (.17)	.42 (.18)	.52 (.16)	.30 (.19)	.34 (.19)	.43 (.20)	.79 (1.10)	1.40 (1.99)	.01 (.00)	1.33 (.00)	.07 (.07)

Note: "C&SE" refers to the Cognitive & Social-emotional Competencies development domain. Numbered columns list average item-level statistics across countries 1 to 12, the numbers within parenthesis are the standard deviations of those statistics. Column 1 refers to the proportion of cases answering incorrectly and ordinal item, column 2 to the proportion of cases answer correctly a dichotomous or an ordinal item, column 3 to the correlation item-domain, column 4 to the correlation item-domain correlation with the item removed from the total domain score, column 5 to the item-test total score correlation, column 6 to the standardized factor loading for item on the latent developmental domain, column 7 to the logistic or ordinal logistic regression coefficient of the child's age on the item probability of being answered correctly, column 8 to the discrimination parameter of the IRT Graded Response Model, column 9 to the difficulty parameter of the IRT Rasch model, and columns 10 and 11 to the discrimination and difficulty parameters of the 2-PL IRT model, respectively.

Table A4. *Average item-level statistics for core items in Child Direct Assessment.*

Item	1	2	3	4	5	6	7	8	9
**Literacy 1**	0.5 (0.2)	0.67 (0.18)	0.49 (0.11)	0.63 (0.13)	0.64 (0.13)	0.1 (0.19)	0.18 (1.51)	2.85 (1.5)	0.1 (0.75)
**Literacy 2**	0.48 (0.3)	0.71 (0.17)	0.47 (0.17)	0.6 (0.15)	0.58 (0.12)	0.07 (0.11)	1.32 (2.51)	3.5 (1.49)	0.68 (1.01)
**Literacy 3**	0.58 (0.26)	0.71 (0.22)	0.51 (0.18)	0.61 (0.13)	0.6 (0.16)	0.05 (0.08)	0.22 (1.37)	3.82 (2.5)	0.31 (0.72)
**Literacy 4**	0.45 (0.24)	0.79 (0.16)	0.59 (0.14)	0.66 (0.11)	0.65 (0.18)	0.06 (0.08)	1.17 (1.53)	4.6 (2.03)	0.72 (0.57)
**Literacy 5**	0.46 (0.27)	0.74 (0.15)	0.59 (0.15)	0.66 (0.16)	0.65 (0.18)	0.06 (0.08)	1.17 (1.73)	4.61 (1.44)	0.69 (0.62)
**Literacy 6**	0.47 (0.27)	0.74 (0.2)	0.55 (0.15)	0.63 (0.13)	0.63 (0.17)	0.05 (0.05)	1.13 (1.73)	4.66 (2.82)	0.67 (0.64)
**Literacy 7**	0.57 (0.29)	0.75 (0.19)	0.53 (0.2)	0.64 (0.12)	0.61 (0.14)	0.07 (0.13)	0.23 (1.72)	4.47 (2.78)	0.38 (0.75)
**Literacy 8**	0.55 (0.17)	0.65 (0.12)	0.51 (0.11)	0.54 (0.08)	0.48 (0.06)	0.08 (0.16)	0.84 (1.05)	3.53 (0.9)	0.7 (0.46)
**Literacy 9**	0.37 (0.2)	0.76 (0.16)	0.64 (0.13)	0.74 (0.11)	0.73 (0.12)	0.1 (0.17)	0.96 (1.35)	3.98 (2.61)	0.48 (0.59)
**Literacy 10**	0.61 (0.15)	0.62 (0.04)	0.43 (0.11)	0.49 (0.07)	0.41 (0.09)	0.11 (0.22)	0.52 (0.63)	2.68 (0.46)	0.65 (0.35)
**Literacy 11**	0.51 (0.21)	0.6 (0.23)	0.51 (0.15)	0.71 (0.12)	0.66 (0.18)	0.1 (0.21)	0.06 (1.27)	3.21 (1.1)	0.03 (0.69)
**Literacy 12**	0.36 (0.18)	0.79 (0.14)	0.66 (0.09)	0.74 (0.14)	0.72 (0.14)	0.15 (0.3)	1.07 (1.36)	3.97 (1.17)	0.52 (0.58)
**Literacy 13**	0.33 (0.2)	0.79 (0.16)	0.7 (0.08)	0.79 (0.08)	0.76 (0.12)	0.13 (0.25)	1.27 (1.4)	5.44 (4.23)	0.6 (0.61)
**Literacy 14**	0.4 (0.26)	0.76 (0.23)	0.55 (0.12)	0.71 (0.11)	0.66 (0.11)	0.11 (0.21)	1.19 (1.72)	3.53 (1.06)	0.68 (0.84)
**Literacy 15**	0.51 (0.16)	0.75 (0.12)	0.56 (0.12)	0.62 (0.1)	0.6 (0.08)	0.08 (0.17)	0.57 (0.85)	4.21 (2.61)	0.49 (0.43)
**Literacy 16**	0.57 (0.25)	0.64 (0.28)	0.51 (0.18)	0.68 (0.12)	0.63 (0.12)	0.11 (0.15)	0.37 (1.68)	3.64 (1.61)	0.27 (0.86)
**Literacy 17**	0.57 (0.22)	0.79 (0.12)	0.58 (0.13)	0.61 (0.1)	0.6 (0.13)	0.04 (0.03)	0.27 (1.3)	4.37 (2.12)	0.41 (0.63)
**Literacy 18**	0.84 (0.11)	0.49 (0.07)	0.28 (0.11)	0.47 (0.08)	0.46 (0.1)	0.09 (0.15)	-2.49 (1.11)	0.9 (0.33)	-2.6 (1.24)
**Literacy 19**	0.64 (0.17)	0.37 (0.19)	0.3 (0.08)	0.45 (0.11)	0.4 (0.16)	0.04 (0.07)	-0.76 (1.05)	0.78 (0.27)	-0.75 (1.4)
**Literacy 20**	0.69 (0.14)	0.45 (0.16)	0.3 (0.1)	0.45 (0.09)	0.42 (0.1)	0.06 (0.07)	-1.15 (0.98)	0.76 (0.33)	-1.38 (0.89)
**Literacy 21**	0.55 (0.16)	0.36 (0.13)	0.31 (0.1)	0.43 (0.08)	0.39 (0.12)	0.03 (0.04)	-0.3 (0.96)	0.7 (0.25)	-0.13 (1.18)
**Literacy 22**	0.37 (0.23)	0.69 (0.12)	0.46 (0.06)	0.57 (0.07)	0.6 (0.08)	0.13 (0.29)	1.02 (1.18)	1.17 (0.48)	0.76 (1.02)
**Literacy 23**	0.3 (0.22)	0.6 (0.12)	0.47 (0.1)	0.61 (0.09)	0.6 (0.14)	0.16 (0.34)	1.69 (1.41)	1.26 (0.49)	1.09 (1.12)
**Literacy 24**	0.28 (0.18)	0.64 (0.2)	0.49 (0.09)	0.6 (0.12)	0.62 (0.15)	0.16 (0.33)	1.85 (1.37)	1.38 (0.57)	1.15 (1)
**Literacy 25**	0.47 (0.2)	0.44 (0.21)	0.35 (0.19)	0.44 (0.21)	0.46 (0.19)	0.01 (0.01)	0.33 (1.23)	0.72 (0.95)	-4.37 (7.09)
**Literacy 26**	0.43 (0.09)	0.55 (0.09)	0.43 (0.11)	0.5 (0.1)	0.55 (0.07)	0.01 (0.01)	0.5 (0.64)	0.87 (0.88)	0.3 (0.22)
**Literacy 27**	0.31 (0.23)	0.51 (0.28)	0.48 (0.23)	0.65 (0.21)	0.56 (0.17)	0.13 (0.14)	1.44 (1.43)	1.43 (0.7)	0.84 (1.15)
**Numeracy 28**	0.59 (0.1)	0.6 (0.1)	0.41 (0.11)	0.48 (0.16)	0.47 (0.16)	0.08 (0.16)	-0.51 (0.6)	0.91 (0.39)	-0.54 (0.6)
**Numeracy 29**	0.5 (0.25)	0.94 (0.06)	0.61 (0.11)	0.75 (0.11)	0.75 (0.11)	0.09 (0.18)	0.63 (1.97)	11.13 (7.48)	0.39 (0.86)
**Numeracy 30**	0.53 (0.26)	0.88 (0.11)	0.56 (0.15)	0.75 (0.14)	0.72 (0.18)	0.11 (0.22)	0.45 (2.05)	4.86 (0.72)	0.36 (0.89)

This is a provisional file, not the final typeset article

**Numeracy 31**	0.73 (0.22)	0.69 (0.27)	0.52 (0.13)	0.75 (0.09)	0.72 (0.08)	0.12 (0.2)	-1.84 (2.01)	3.41 (1.05)	-0.81 (0.94)
**Numeracy 32**	0.63 (0.25)	0.87 (0.08)	0.63 (0.07)	0.77 (0.11)	0.79 (0.08)	0.08 (0.1)	-0.74 (1.93)	4.2 (1.26)	-0.24 (0.87)
**Numeracy 33**	0.6 (0.24)	0.88 (0.11)	0.67 (0.08)	0.81 (0.08)	0.82 (0.05)	0.14 (0.24)	-0.58 (1.89)	6.42 (7.05)	-0.21 (0.84)
**Numeracy 34**	0.68 (0.24)	0.83 (0.12)	0.62 (0.1)	0.77 (0.06)	0.74 (0.08)	0.11 (0.17)	-1.29 (1.91)	4.05 (0.98)	-0.53 (0.88)
**Numeracy 35**	0.67 (0.23)	0.8 (0.19)	0.61 (0.09)	0.79 (0.07)	0.78 (0.07)	0.12 (0.2)	-1.29 (1.83)	3.86 (1.06)	-0.53 (0.83)
**Numeracy 36**	0.56 (0.17)	0.91 (0.07)	0.65 (0.06)	0.76 (0.06)	0.75 (0.11)	0.09 (0.18)	0.05 (1.32)	7.17 (4.52)	0.17 (0.74)
**Numeracy 37**	0.54 (0.26)	0.95 (0.07)	0.63 (0.1)	0.79 (0.11)	0.76 (0.15)	0.04 (0.03)	0.21 (1.72)	9.29 (5.78)	0.26 (0.81)
**Numeracy 38**	0.55 (0.2)	0.92 (0.1)	0.63 (0.09)	0.77 (0.06)	0.73 (0.11)	0.11 (0.21)	0.16 (1.39)	9.98 (7.62)	0.22 (0.73)
**Numeracy 39**	0.64 (0.18)	0.79 (0.08)	0.61 (0.09)	0.72 (0.05)	0.65 (0.12)	0.13 (0.19)	-0.94 (1.32)	2.12 (0.39)	-0.49 (0.71)
**Numeracy 40**	0.7 (0.06)	0.75 (0.08)	0.54 (0.13)	0.67 (0.12)	0.69 (0.11)	0.03 (0.01)	-1.17 (0.55)	1.83 (0.4)	-0.73 (0.31)
**Numeracy 41**	0.49 (0.2)	0.75 (0.1)	0.56 (0.08)	0.7 (0.1)	0.67 (0.08)	0.12 (0.24)	0.26 (1.31)	1.96 (0.45)	0.18 (0.79)
**Numeracy 42**	0.74 (0.1)	0.61 (0.16)	0.46 (0.06)	0.62 (0.11)	0.59 (0.09)	0.06 (0.06)	-1.52 (0.82)	1.34 (0.27)	-1.09 (0.62)
**Numeracy 43**	0.57 (0.14)	0.65 (0.11)	0.54 (0.06)	0.65 (0.08)	0.64 (0.05)	0.1 (0.16)	-0.33 (0.92)	1.45 (0.32)	-0.24 (0.64)
**Numeracy 44**	0.55 (0.13)	0.65 (0.1)	0.54 (0.07)	0.67 (0.07)	0.67 (0.08)	0.06 (0.06)	-0.27 (0.72)	1.51 (0.37)	-0.16 (0.51)
**Numeracy 45**	0.36 (0.14)	0.69 (0.13)	0.59 (0.06)	0.65 (0.06)	0.7 (0.03)	0.33 (0.54)	0.92 (0.77)	1.88 (0.41)	0.53 (0.64)
**Numeracy 46**	0.74 (0.21)	0.42 (0.44)	0.53 (0.03)	0.6 (0.15)	0.64 (0.23)	0.34 (0.41)	-1.53 (1.46)	1.26 (0.29)	-1.31 (1.39)
**Numeracy 47**	0.35 (0.09)	0.46 (0.21)	0.29 (0.18)	0.4 (0.16)	0.39 (0.23)	0 (0.03)	0.92 (0.56)	0.62 (0.35)	-12.87 (33.91)
**Numeracy 48**	0.4 (0.06)	0.42 (0.07)	0.26 (0.06)	0.33 (0.1)	0.31 (0.08)	0.01 (0.02)	0.54 (0.34)	0.48 (0.24)	1.72 (2.49)
**Numeracy 49**	0.55 (0.1)	0.59 (0.14)	0.41 (0.05)	0.47 (0.09)	0.45 (0.1)	0.02 (0.02)	-0.29 (0.58)	0.85 (0.26)	-0.26 (0.45)
**Numeracy 50**	0.65 (0.15)	0.64 (0.23)	0.46 (0.21)	0.5 (0.2)	0.52 (0.21)	0.11 (0.18)	-0.89 (1.15)	1.11 (0.6)	-0.58 (0.76)
**Numeracy 51**	0.61 (0.14)	0.67 (0.04)	0.56 (0.14)	0.55 (0.08)	0.58 (0.12)	0.15 (0.27)	-0.54 (1.1)	1.12 (0.33)	-0.52 (0.89)
**Numeracy 52**	0.59 (0.27)	0.64 (0.08)	0.49 (0.06)	0.5 (0.07)	0.51 (0.03)	0.13 (0.17)	-0.41 (1.97)	1 (0.17)	-0.43 (1.49)
**Numeracy 53**	0.82 (0.09)	0.51 (0.14)	0.39 (0.11)	0.49 (0.09)	0.47 (0.1)	0.07 (0.13)	-2.23 (0.86)	1 (0.24)	-1.9 (0.58)
**Numeracy 54**	0.73 (0.13)	0.55 (0.13)	0.4 (0.09)	0.46 (0.11)	0.42 (0.11)	0.04 (0.05)	-1.54 (0.98)	0.94 (0.37)	-1.54 (1.39)
**Numeracy 55**	0.64 (0.15)	0.6 (0.1)	0.43 (0.08)	0.5 (0.07)	0.47 (0.06)	0.06 (0.12)	-0.61 (0.59)	0.91 (0.22)	-0.56 (0.47)
**Numeracy 56**	0.61 (0.06)	0.59 (0.12)	0.46 (0.04)	0.51 (0.08)	0.47 (0.06)	0.08 (0.15)	-0.62 (0.38)	0.96 (0.17)	-0.56 (0.36)
**EF 57**	0.51 (0.09)	0.72 (0.19)	0.53 (0.15)	0.55 (0.14)	0.4 (0.46)	0.04 (0.06)	0.59 (0.66)	2.64 (1.24)	0.27 (0.49)
**EF 58**	0.52 (0.13)	0.83 (0.17)	0.55 (0.15)	0.58 (0.1)	0.4 (0.51)	0.05 (0.07)	0.54 (0.9)	2.99 (1.31)	0.16 (0.77)
**EF 59**	0.49 (0.12)	0.79 (0.17)	0.54 (0.15)	0.57 (0.11)	0.38 (0.47)	0.04 (0.04)	0.74 (0.78)	3.1 (1.49)	0.24 (0.72)
**EF 60**	0.52 (0.1)	0.79 (0.2)	0.53 (0.18)	0.55 (0.13)	0.34 (0.45)	0.04 (0.04)	0.57 (0.72)	3.3 (1.95)	0.04 (1.01)
**EF 61**	0.52 (0.08)	0.86 (0.18)	0.57 (0.18)	0.58 (0.12)	0.36 (0.5)	0.04 (0.04)	0.56 (0.58)	3.52 (1.88)	0.2 (0.53)
**EF 62**	0.5 (0.13)	0.82 (0.19)	0.55 (0.17)	0.57 (0.1)	0.39 (0.5)	0.05 (0.08)	0.71 (0.87)	3.04 (1.56)	0.19 (0.84)

**EF 63**	0.59 (0.28)	0.85 (0.17)	0.46 (0.14)	0.57 (0.25)	0.51 (0.23)	0.09 (0.15)	0.11 (3.17)	2.34 (1.52)	-1.34 (2.48)
**EF 64**	0.47 (0.27)	0.65 (0.22)	0.47 (0.18)	0.49 (0.3)	0.5 (0.18)	0.06 (0.09)	0.86 (2.8)	2.02 (1.51)	-1.19 (2.87)
**EF 65**	0.51 (0.24)	0.77 (0.18)	0.48 (0.14)	0.47 (0.37)	0.49 (0.15)	0.08 (0.14)	0.85 (3.05)	3.3 (2.41)	2.87 (8.02)
**EF 66**	0.52 (0.24)	0.8 (0.19)	0.48 (0.15)	0.55 (0.23)	0.48 (0.18)	0.09 (0.16)	0.82 (3.07)	3.35 (2.73)	-1.99 (4.83)
**EF 67**	0.56 (0.12)	0.66 (0.25)	0.46 (0.15)	0.5 (0.19)	0.36 (0.4)	0.03 (0.05)	0.32 (0.84)	1.92 (0.9)	0.1 (0.72)
**EF 68**	0.5 (0.16)	0.65 (0.18)	0.47 (0.13)	0.49 (0.16)	0.36 (0.38)	0.01 (0.03)	0.69 (1.07)	1.97 (0.91)	0.27 (0.97)
**EF 69**	0.59 (0.04)	0.75 (0.13)	0.63 (0.15)	0.64 (0.19)	0.71 (0.16)	0.04 (0.04)	-0.57 (0.27)	2.06 (0.27)	-0.28 (0.13)
**EF 70**	0.62 (0.07)	0.85 (0.09)	0.66 (0.17)	0.62 (0.23)	0.69 (0.21)	0.04 (0.03)	-0.77 (0.57)	2.82 (0.87)	-0.36 (0.29)
**EF 71**	0.59 (0.05)	0.85 (0.1)	0.67 (0.14)	0.68 (0.14)	0.73 (0.1)	0.05 (0.04)	-0.52 (0.21)	2.75 (0.76)	-0.25 (0.1)
**EF 72**	0.58 (0.07)	0.84 (0.11)	0.67 (0.14)	0.7 (0.16)	0.74 (0.11)	0.06 (0.05)	-0.45 (0.37)	2.7 (0.5)	-0.22 (0.15)
**EF 73**	0.59 (0.06)	0.79 (0.17)	0.63 (0.17)	0.56 (0.21)	0.6 (0.15)	0.04 (0.03)	-0.59 (0.43)	2.64 (1.08)	-0.29 (0.25)
**EF 74**	0.62 (0.06)	0.84 (0.08)	0.64 (0.13)	0.6 (0.2)	0.64 (0.16)	0.04 (0.04)	-0.75 (0.47)	3.08 (1.46)	-0.34 (0.26)
**EF 75**	0.57 (0.05)	0.82 (0.12)	0.68 (0.16)	0.7 (0.17)	0.76 (0.12)	0.06 (0.05)	-0.38 (0.11)	2.53 (0.64)	-0.19 (0.06)
**EF 76**	0.69 (0.07)	0.86 (0.03)	0.65 (0.09)	0.71 (0.1)	0.71 (0.04)	0.06 (0.04)	-1.26 (0.36)	2.79 (0.41)	-0.55 (0.18)
**EF 77**	0.64 (0.07)	0.86 (0.15)	0.69 (0.16)	0.71 (0.17)	0.79 (0.14)	0.06 (0.05)	-0.87 (0.37)	3.47 (1.45)	-0.39 (0.16)
**EF 78**	0.63 (0.07)	0.89 (0.1)	0.68 (0.12)	0.69 (0.19)	0.74 (0.13)	0.06 (0.05)	-0.84 (0.38)	2.93 (0.5)	-0.37 (0.17)
**EF 79**	0.65 (0.06)	0.86 (0.14)	0.63 (0.16)	0.6 (0.29)	0.61 (0.24)	0.06 (0.05)	-0.98 (0.47)	2.71 (0.41)	-0.43 (0.21)
**EF 80**	0.64 (0.08)	0.84 (0.17)	0.64 (0.2)	0.62 (0.29)	0.6 (0.22)	0.05 (0.05)	-0.89 (0.58)	2.98 (0.77)	-0.4 (0.26)
**EF 81**	0.34 (0.16)	0.52 (0.32)	0.4 (0.13)	0.52 (0.18)	0.43 (0.4)	0.03 (0.04)	2.06 (1.35)	1.14 (0.75)	1.42 (1.07)
**EF 82**	0.55 (0.14)	0.57 (0.22)	0.46 (0.15)	0.52 (0.08)	0.52 (0.41)	0.05 (0.04)	-0.26 (0.95)	1.03 (0.47)	-0.2 (0.75)
**EF 83**	0.29 (0.16)	0.55 (0.26)	0.48 (0.15)	0.5 (0.08)	0.49 (0.37)	0.03 (0.03)	1.58 (1.21)	2.25 (4.09)	1.29 (1.11)
**SE 84**	0.74 (0.09)	0.9 (0.08)	0.45 (0.16)	0.48 (0.11)	0.66 (0.08)	0.07 (0.09)	-1.68 (0.83)	1.29 (0.42)	-1.13 (0.46)

Note: "EF" refers to the Executive Functioning Developmental Domain and "SE" refers to the Social-emotional Competencies development domain. Numbered columns list average item-level statistics across countries 1 to 9, the numbers within parenthesis are the standard deviations of those statistics. Column 1 refers to the proportion of cases answer correctly a dichotomous or an ordinal item, column 2 to the correlation item-domain, column 3 to the correlation item-domain correlation with the item removed from the total domain score, column 4 to the item-test total score correlation, column 5 to the standardized factor loading for item on the latent developmental domain, column 6 to the logistic or ordinal logistic regression coefficient of the child's age on the item probability of being answered correctly, column 7 to the difficulty parameter of the IRT Rasch model, columns 8 and 9 to the discrimination and difficulty parameters of the 2-PL IRT model, respectively.

This is a provisional file, not the final typeset article

Table B1. ***Content overlap between core set of items and other child development instruments.***

**Domains**	**Number of core items**	**Number of core items present in:**			
		**eHCI**	**IDELA**	**PRIDI**	**EAP-ECDS**
Caregiver report					
**Early Literacy**	5	5	*NA*	1	0
**Early Numeracy**	6	6	*NA*	1	1
**EF & Social-emotional competencies**	9	5	*NA*	2	2
**Direct assessment**					
**Early Literacy**	27	*NA*	25	0	13
**Early Numeracy**	29	*NA*	19	11	7
**Executive Functioning**	27	*NA*	7	1	6
**Social-emotional competencies**	1	*NA*	1	0	1

Note: "EF" refers to the Executive Functioning Developmental Domain. "eHCI" stands for the early Human Capability Index (Sincovich, Gregory, Zanon, Santos, Lynch, & Brinkman, 2019), "IDELA" for the International Development and Early Learning Assessment (Pisani, Borisova, & Dowd, 2015), PRIDI for the Regional Project on Child Development Indicators (Verdisco et al, 2015), and EAP-ECDS for the East Asia-Pacific Early Child Development Scales (Rao et al., 2014). IDELA includes only a direct assessment of children, eHCI only includes a caregiver report, PRIDI and EAP-ECDS are primarily direct assessment tools, but have accompanying CGV questionnaires.
